# Prediction of arterial blood gas values from arterialized earlobe blood gas values in patients treated with mechanical ventilation

**DOI:** 10.4103/0972-5229.43677

**Published:** 2008

**Authors:** Azim Honarmand, Mohammadreza Safavi

**Affiliations:** **From:** Department of Anaesthesiology and Intensive Care, Isfahan University of Medical Sciences, Isfahan, Iran

**Keywords:** Arterialized earlobe blood gas, critically illness, mechanical ventilation

## Abstract

**Background/Objective:**

Arterial blood gas (ABG) analysis is useful in evaluation of the clinical condition of critically ill patients; however, arterial puncture or insertion of an arterial catheter may sometimes be difficult and cause many complications. Arterialized ear lobe blood samples have been described as adequate to gauge gas exchange in acute and chronically ill pediatric patients.

**Purpose:**

This study evaluates whether pH, partial pressure of oxygen (*P*O_2_), partial pressure of carbon dioxide (*P*CO_2_), base excess (BE), and bicarbonate (HCO_3_) values of arterialized earlobe blood samples could accurately predict their arterial blood gas analogs for adult patients treated by mechanical ventilation in an intensive care unit (ICU).

**Setting:**

A prospective descriptive study

**Methods:**

Sixty-seven patients who were admitted to ICU and treated with mechanical ventilation were included in this study. Blood samples were drawn simultaneously from the radial artery and arterialized earlobe of each patient.

**Results:**

Regression equations and mean percentage-difference equations were derived to predict arterial pH, *P*CO_2_, *P*O_2_, BE, and HCO_3_-values from their earlobe analogs. pH, *P*CO_2_, BE, and HCO_3_ all significantly correlated in ABG and earlobe values. In spite of a highly significant correlation, the limits of agreement between the two methods were wide for *P*O_2_. Regression equations for prediction of pH, *P*CO_2_, BE, and HCO3- values were: arterial pH (pHa) = 1.81+ 0.76 × earlobe pH (pHe) [r = 0.791, *P* < 0.001]; PaCO_2_ = 1.224+ 1.058 × earlobe*P*CO_2_ (PeCO_2_) [r = 0.956, *P* < 0.001]; arterial BE (BEa) = 1.14+ 0.95 × earlobe BE (BEe) [r= 0.894, *P* < 0.001], and arterial HCO_3_- (HCO_3_-a) = 1.41+ earlobe HCO_3_(HCO_3_-e) [r = 0.874, *P* < 0.001]. The predicted ABG values from the mean percentage-difference equations were derived as follows: pHa = pHe × 1.001; PaCO_2_ = PeCO_2_ × 0.33; BEa = BEe × 0.57; and HCO_3_-a = HCO_3_-e × 1.06.

**Conclusions:**

Arterialized earlobe blood gas can accurately predict the ABG values of pH, *P*CO_2_, BE, and HCO_3_- for patients who do not require regular continuous blood pressure measurements and close monitoring of arterial *P*O_2_ measurements.

## Introduction

Measurement of arterial blood gas tensions is routinely used to assess gas exchange in patients with acute and chronic respiratory disorders. Arterial blood gas (ABG) sampling represents the gold standard method for acquiring patients' acid-base status. The most common complications associated with arterial puncture are pain, arterial injury, aneurysm formation, hemorrhage, and thrombosis with distal ischaemia. The risks increase with repeated arterial punctures, especially with insertion of a catheter when performed by inexperienced individuals.[1] Additionally, this procedure carries a small but appreciable risk of needle stick injury to health care workers, with the consequent risk of transmission of blood borne viruses such as hepatitis C and human immunodeficiency virus (HIV).[2] In the 1960s, it was proposed that blood gas values could be measured using arterialized earlobe blood samples.[3] Arterialized ear lobe blood gas samplings are easier to obtain and a less invasive way of evaluating acid-base status in intensive care unit. It avoids the risks of arterial punctures. It is based on the assumption that provided sufficient vasodilatation can be achieved locally by means of topical application of a vaso-active cream on the earlobe, the arterialized earlobe oxygen tension resembles the arterial oxygen tension due to convergence of arterial and venous oxygen tension.[4] Techniques for sampling arterialized capillary blood from the finger pulp and the earlobe were first described over two decades ago but, although close agreement between arterial values and earlobe samples has been demonstrated in normal subjects, this technique is not in common usage. The main reasons for this appear to be lack of knowledge of its existence and uncertainty over its accuracy.[5] Initial[3,6,7] or more recent[5] studies have concluded that the earlobe method might be accurate enough to replace arterial blood samples for clinical purposes. This opinion is based mainly on positive and strong correlations that have been found between the two methods. However, most investigators have sought correlation using simple regression analysis instead of the method of BLAND and ALTMAN.[8] This approach has probably rendered the conclusion of most studies flawed by statistical bias. In fact, two recent studies using BLAND and ALTMAN[8] analysis have stated that arterialized earlobe *P*O_2_ often underestimates arterial *P*O_2_, therefore making this method unsuitable for clinical assessment.[9,10] Several studies have shown good correlation between capillary blood, venous blood, and arterial blood gas values in pediatric intensive care units.[11–13] However, arterialized earlobe oxygen tension often underestimates arterial oxygen tension[4] and is not fully validated in adult patients with acute respiratory failure receiving mechanical ventilation. The purpose of this study was to investigate the correlation between simultaneous arterial blood gas and arterialized earlobe blood samples and to establish whether pH, partial pressure of oxygen (*P*O_2_), partial pressure of carbon dioxide (*P*CO_2_), base excess (BE), and bicarbonate (HCO_3_-) values of arterialized earlobe blood samples could accurately predict their arterial blood gas analogs for patients with acute respiratory failure treated by mechanical ventilation in an intensive care unit (ICU).

## Materials and Methods

Sixty seven patients with acute respiratory failure who were admitted to a multidisciplinary adult intensive care unit between May 2005 and August 2005 receiving mechanical ventilation, were enrolled in this prospective descriptive study. The study protocol was approved by the local institutional ethics committee, and written informed consent was obtained from each patient or his or her family. Patients with hypotension (systolic blood pressure less than or equal to 90 mm Hg), hypertension (systolic pressure above 140 with a diastolic pressure above 90), hypothermia (axillary temperature <36°C), and hyperthermia (axillary temperature >38°C), severe sepsis, multiorgan failure, or chronic lung disease were excluded from the study. No patient was in cardiovascular shock.

Blood samples were drawn simultaneously from the radial artery and the arterialized earlobe of each patient in a sitting position. Blood gases were obtained if the patient needed blood gases for clinical decisions. All patients were receiving mechanical ventilation for 48h or more with a fraction of inspired oxygen (*F*iO_2_) of 0.5 or less and positive end-expiratory pressure of 5 cm H_2_O.

### Arterial samples

If the patient had an arterial line, they were used for blood sampling. After withdrawing 5 ml of blood from the lines with a non-heparinised syringe, 1 ml of arterial blood was obtained with another similar syringe and transferred, as soon as possible, to a heparinized 0.75 ml capillary tube. If the patient did not have an arterial line, arterial punctures with aseptic precautions were carried out, and blood was transferred directly into an identical capillary tube.

### Earlobe samples

After aseptic cleaning, the lateral distal portion of the earlobe was punctured with a scalpel blade (surgical blade No. 11, Troge®Blades, Hamburge, Germany) and blood gases samples were obtained by “contact” with the capillary tube's tip. A short manual massage was necessary in some instances. Two sides of the tube were closed by fingers to avoid air bubbles. All samples were obtained by the investigators. Arterialized samples were collected in heparinized glass capillaries (Modulohm A/S, Vasekaer, Herlev, Denmark) and immediately introduced into the blood gas analyzer (AVL Compact 3, Roche Diagnostics GmbH, Mannheim, Germany) followed within 2 min by arterial samples. pH, *P*O_2_, *P*CO_2_, BE, and HCO_3_ values were recorded.

### Statistical methods

The statistical analysis for assessing agreement between arterial and arterialized blood gases was performed according to BLAND and ALTMAN.[8] SPSS.11.5 for Windows was used for statistical analysis. Pearson correlation coefficients were determined for correlation and regression equations and mean percentage-difference equations were derived to predict arterial pH, *P*CO_2_, BE, and HCO_3_ - values from their arterialized ear lobe blood gas analogs. For all analyses, *P*<0.05 was considered statistically significant.

## Results

**Figure 1:**
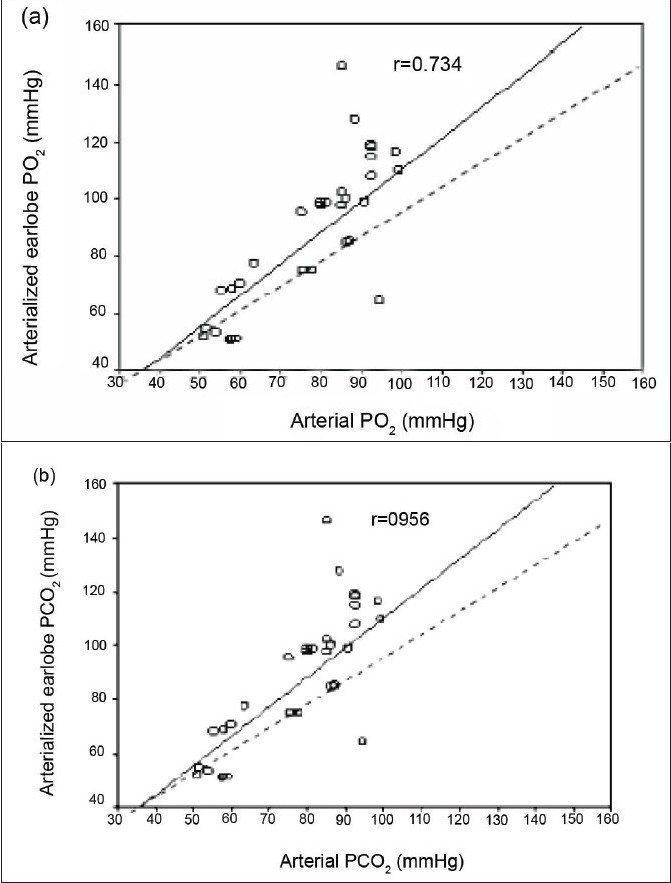
Correlation between radial artery and and arterialized earlobe blood gas, a) Partial pressure of oxygen (PO2) values, b) Partial pressure of carbon dioxide (PCO2) values …………., line of identity: ———— regression lines

**Figure 2:**
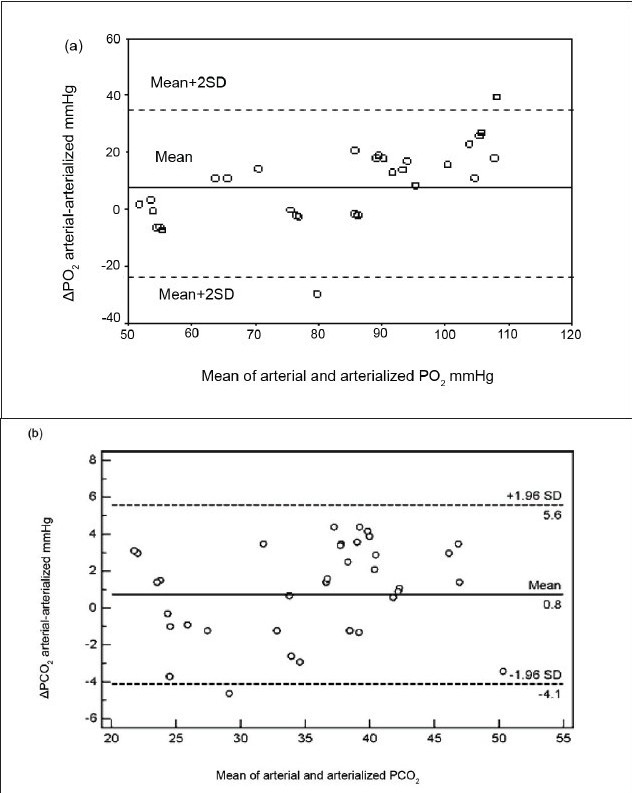
a) Differences in arterial –arterialized partial pressure of oxygen values (ΔPO_2_) plotted against mean of arterial and arterialized PO_2_ values. b) Differences in arterial-arterialized partial pressure of carbon dioxide values (ΔPCO_2_) plotted against mean of arterial and arterialized PCO_2_ values

Sixty-seven consecutive adult patients were studied. Blood samples were drawn simultaneously from arterialized earlobe and radial artery. Admission diagnoses included head trauma, abdominal mass, gastrointestinal bleeding, lung contusion, bowel obstruction, myasthenia gravis, epilepsy, pneumonia, meningitis, diabetic ketoacidosis, stroke, brain tumor, lung cancer, and intoxication [Table 1]. The mean (SD) age of the patients was 47.57 (19.51). Fifty (74.6%) were males and 17 (25.4%) were females. No complication in the drawing of blood samples was observed with either method. pH, *P*CO2, BE, and HCO3 were all significantly correlated in ABG and earlobe samples [Tables 2 and 3]. The range of arterial *P*O2 values was 6.8–19.5 kPa (51.1–146.5mmHg), mean 11.3 kPa (84.77mmHg). The range and mean of arterial *P*CO_2_ values was 3.01–6.5 kPa (22.6–48.6 mmHg) and 4.7 kPa (35.08 mmHg) respectively. The relationships between arterial and earlobe samples for *P*O_2_ and *P*CO2 are shown in [Figure 1a and b]. The correlation coefficients were 0.734 (*P*<0.0001) and 0.956 (*P*<0.0001) respectively. Despite this highly significant correlation, regression lines were slightly different from lines of identity, particularly for *P*O_2_. In Figure 2a and b, differences between the two methods (arterial - arterialized values) were plotted against means of arterial and arterialized *P*O_2_PO_2_ or *P*CO_2_. The mean (bias) ±SD and the range of the differences, as well as the 95% confidence intervals for the lower and upper limit of agreement were reported in Table 4. Arterialized earlobe *P*O_2_ was lower than arterial *P*O_2_ in most cases, and the difference increased as the arterial *P*O_2_ increased. These results show that the limits of agreement for *P*O_2_ were wide, reveal a lack of agreement between the two methods. For *P*CO_2_, on the other hand, the mean difference between the two methods was close to zero, and the limits of agreement were narrower. Regression equations for prediction of pH, *P*CO_2_,, BE, and HCO_3_ - values were: arterial pH (pHa) = 1.81+ 0.76 × earlobe pH (pHe) [r = 0.791, *P* < 0.001]; *P*CO_2_, = 1.224+ 1.06 × earlobe *P*CO_2_, (PeCO_2_) [r = 0.956, *P* < 0.001]; arterial BE (BEa) = 1.14+ 0.95 × earlobe BE (BEe) [r= 0.894, *P* < 0.001], and arterial HCO_3_- (HCO_3_-a) = 1.41+ earlobe HCO_3_ (HCO_3_-e) [r = 0.874, *P* < 0.001]. The predicted ABG values from the mean percentage-difference equations were derived as follows: pHa = pHe × 1.001; *P*CO_2_, = PeCO_2_ × 0.33; BEa = BEe × 0.57; and HCO_3_-a = HCO_3_-e × 1.06.

**Table 1:** Clinical diagnoses of the patients

Diagnosis	n (%)
Head trauma	27 (40)
Stroke	11 (16.5)
Pneumonia	8 (12)
Epilepsy	4 (6)
Gastrointestinal bleeding	3 (4.5)
Bowel obstruction	3 (4.5)
Abdominal mass	2 (3)
Lung contusion	2 (3)
Meningitis	2 (3)
Myasthenia gravis	1 (1.5)
Diabetic ketoacidosis	1 (1.5)
Brain tumor	1 (1.5)
Lung cancer	1 (1.5)
Intoxication	1 (1.5)
Total	67 (100)

**Table 2:** Correlation of arterial and arterialized earlobe blood samples for pH, PO_2_, PCO_2_, BE, and HCO_3_ in patients

	pH		PO_2_		PCO_2_		BE		HCO_3_	
					
	Arterial	Earlobe	Arterial	Earlobe	Arterial	Earlobe	Arterial	Earlobe	Arterial	Earlobe
Arterial	1.000	0.791	1.000	0.734	1.000	0.774	1.000	0.894	1.000	0.874	
	*P*<0.001		*P* <0.001		*P* <0.001		*P* <0.001		*P* <0.001	
Earlobe	0.791	1.000	0.734	1.000	0.774	1.000	0.894	1.000	0.874	1.000	
	*P*<0.001		*P* <0.001		*P* <0.001		*P* <0.001		*P* <0.001	

**Table 3:** Regression of arterial blood gas values on arterialized earlobe blood samples values

Arterial	Earlobe Constant (SE)	β (SE)	R^2^	SE of estimate
pH	1.81 (0.537)	0.757 (0.073)	0.626	0.029
PCO_2_	11.438 (2.483)	0.703 (0.071)	0.599	5.271
PO_2_	10.522 (9.877)	1.093 (0.126)	0.538	16.199
BE	1.138 (0.317)	0.952 (0.059)	0.800	2.183
HCO_3_	1.406 (1.438)	0.996 (0.069)	0.764	2.644

**Table 4:** Limits of agreement of the differences in *P*O_2_ and *P*CO_2_ values between arterial and arterialized earlobe blood samples

	Δ*P*O_2_		Δ*P*CO_2_	
		
	kPa	mmHg	kPa	mmHg
Mean±SD	1.02±2.15	7.7±16.14	0.19±0.78	1.45±5.88
Range	0.04-6.32	0.3-47.4	-3.52-0.32	-26.5-2.4
95% CI of				
mean+ 2SD	3.78-6.89	28.35-51.61	1.37-2.15	10.33-16.09
mean - 2SD	-4.83− -1.68	-36.21− -12.59	-1.77− -0.99	-13.19− -7.43

Δ*P*O_2_: Difference in partial pressure of oxygen (arterial-arterialized); Δ*P*CO_2_: Difference in partial pressure of carbon dioxide (arterial-arterialized). 95% CI: 95% confidence interval. (1 mmHg=133.32 Pa)

## Discussion

Acid-base analysis is essential for management of patients in ICU, yielding valuable information about a variety of disease processes.[1,12,14] Non-invasive methods, such as pulse oximetry, transcutaneous monitoring of oxygen and carbon dioxide, and end tidal carbon dioxide have been proven to be useful,[1] but they do not give information about pH, *P*O_2_, BE and bicarbonate.

Arterial blood gases are frequently determined in the ICU. This is, however, an invasive way of monitoring blood gas and there are complications, mostly local hematoma related to arterial puncture. The procedure itself is technically difficult and painful.

Arterial lines are usually placed in unstable patients in the ICU who need close monitoring of *P*O2. When an arterial line is not in place, arterial or venous blood gas (VBG) values continue to be obtained and used for clinical monitoring and management decisions.

Earlobe blood gas sampling is a less invasive way of evaluating acid-base status in a well perfused patient. If a blood gas value determined by earlobe samples could be used to show patients' acid-base status and guide their management with the same accuracy as arterial sampling, this would be preferable because of ease of blood sample collection.

For many years, clinicians have been looking for alternatives to ABG sampling in both children and adults, and studies have investigated ABG, VBG, and capillary (CBG) blood gas samples and the correlation between the values.[15–17]

Numerous studies published many years ago concluded that the method using arterialized earlobe blood for *P*O_2_ and *P*CO_2_ analysis was accurate enough to replace arterial blood samples for clinical purposes.[3,6,7,18–20] However, the validity of this method has been discussed in two studies,[9,10] showing with the analysis of BLAND and ALTMAN,[8] *P*O_2_ was usually lower in earlobe than in arterial blood, and that the limits of agreement were wide between the two methods.

Studies comparing ABG and CBG, ABG and VBG samples in diabetic ketoacidosis, and ABG–CBG blood gases values in stable pediatric intensive care unit patients have shown good correlation among ABG, VBG, and CBG samples.[13] However, there have been no studies comparing simultaneously obtained ABG and earlobe samplings in stable adult ICU patients treated by mechanical ventilation.

We studied sixty-seven simultaneously obtained arterial blood gas and arterialized earlobe blood samples of patients and showed pH, *P*CO_2_, BE, HCO_3_ were all correlated in arterial and earlobe blood gases in normotensive and normothermic patients. Although there was a significant correlation for *P*O_2_ in these patients, it was lower.

Sauty *et al*, compared arterial and arterialized earlobe blood samples in 115 consecutive adult patients and concluded that, in adult patients, arterialized earlobe blood *P*O_2_ is not a reliable mirror of arterial *P*O_2_.[9] The main cause of underestimation of arterial *P*O_2_ in earlobe samples is insufficient arterialization of blood, corresponding to a certain venous admixture. The effect of a given venous admixture in earlobe blood depends on the arterio-venous *P*O_2_ difference: the larger the arterio-venous *P*O_2_ difference, the wider the discrepancy between earlobe and arterial *P*O_2_. This is one likely reason for the unreliable *P*O_2_ values measured in arterialized earlobe blood in patients breathing 100% oxygen.[19,21,22]

Because the arterio-venous *P*O_2_ difference is large in subjects with normal arterial *P*O_2_, a small venous admixture in earlobe blood will result in a greater discrepancy between earlobe and arterial *P*O_2_. Our data supports this observation.

It must be emphasized that in our study, there was no patient with severe sepsis, multiorgan failure, cardiovascular shock, or chronic lung disease because inclusion of these patients might have caused poorer correlation between ear and arterial *P*O_2_values due to venous admixture.

Indeed, [Figure 2a] shows that arterial *P*O_2_, when in the normal range, was often markedly underestimated by the earlobe sample. Interestingly and despite fewer normal *P*O_2_ values, the study of PITKIN *et al*,[5] also showed a trend towards increased difference between arterial and arterialized *P*O_2_ with increasing mean *P*O_2_ values. Accordingly, and as reported by the same authors, we observed a better agreement between the two methods for arterial *P*O_2_ values lower than 8.0 kPa (60 mmHg), where the effect of venous admixture is smaller.

On the other hand, there was a good agreement between earlobe and arterial values of *P*CO_2_, as previously reported.[5] This reflects the insignificant effect of venous admixture, due to the comparatively smaller arterio-venous *P*CO_2_ difference.

The usefulness of each method should be weighed according to its advantages and inconveniences. The advocated advantages of the earlobe method are that it is safe and can be performed by non-medical staff.[23] However, as the collection of an earlobe blood sample must be fully aseptic,[6] the method requires trained personnel.

Although complications of arterial punctures have been described,[24] complications of radial arterial punctures are extremely low and in our experience none were observed in the present experiment.

In conclusion, we showed a good correlation in pH, *P*CO_2_, BE, and HCO_3_ between simultaneous samples of arterialized earlobe and arterial blood in normotensive and normothermic patients receiving mechanical ventilation. So, earlobe blood gas measurements may be useful alternatives to arterial blood gas samples for critically ill patients who do not require regular continuous blood pressure measurements and close monitoring of arterial *P*O_2_ measurements. We do not recommend arterialized earlobe blood samples for determining *P*O_2_ of arterial blood gas samples.
